# Genetic characteristics of novel extreme alkaline-inducible promoter located in five prime upstream region of peptidyl-prolyl cis/trans isomerase from *Vibrio anguillarum*

**DOI:** 10.1038/s41598-025-98559-y

**Published:** 2025-07-01

**Authors:** Dong-Gyun Kim, Gyu Min Kim, Dong Nyoung Oh, Young-Sam Kim, Jong Min Lee

**Affiliations:** 1 Biotechnology Research Division, National Institute of Fisheries Science, Busan, Republic of Korea; 2 Department of Biotechnology, Pukyong National University, Busan, 48513 Republic of Korea

**Keywords:** Alkaline-inducible promoter, *Vibrio anguillarum*, Peptidyl-prolyl Cis/trans isomerase, Synthetic biology, Biofoundry, Microbiology, Molecular biology

## Abstract

This study presents the identification and characterization of the promoter region of *Vibrio anguillarum* NB10, which enhances the expression of FK506-binding protein (FKBP)-type peptidyl-prolyl cis/trans isomerase (PPIase; FklB), capable of binding to the immunosuppressant FK506 under extremely alkaline conditions. Our proteomic analysis of *V*. *anguillarum* NB10 revealed that FklB (VaFklB) expression is significantly upregulated under extreme alkaline stress (pH 10). When the putative core promoter regions were coupled with a β-galactosidase reporter gene and introduced into *Escherichia coli*, we observed β-galactosidase activities of 61.47 ± 2.91 and 95.83 ± 6.76 Miller units (MU) at pH 9 and 10, respectively, after 4 h of stress exposure. These values represent 1.97- and 2.88-fold increases compared to normal conditions (25 °C, pH 7: 31.27 ± 1.15 MU). This alkaline-inducible promoter system has potential for biotechnological applications, including the development of pH-responsive gene expression systems, biomanufacturing processes requiring alkaline environments, and targeted activation of silent biosynthetic gene clusters for novel bioactive compound discovery. Our findings provide a valuable molecular tool for synthetic biology and metabolic engineering, enabling precise genetic control under specific environmental conditions that may revolutionize industrial biotechnology.

**Supplementary Information:**

The online version contains supplementary material available at 10.1038/s41598-025-98559-y.

## Introduction

The dynamic regulation of gene expression in response to environmental cues represents a critical adaptation mechanism in bacteria, and harnessing these regulatory systems offers powerful tools for synthetic biology and metabolic engineering. While extensive research has characterized various stress-responsive promoters, including those activated under acidic conditions, osmotic stress, and temperature fluctuations, there remains a significant knowledge gap regarding promoters specifically induced under extremely alkaline conditions^1^. This limitation constrains our ability to develop microbial cell factories for biotechnological applications in alkaline environments, such as bioremediation of alkaline industrial waste, enzyme production for detergent formulations, and expression of therapeutic proteins requiring alkaline pH for optimal activity.

The evolutionary history of marine organisms extends back more than 3 billion years, and marine ecosystems comprise more than three times the number of phyla found in terrestrial ecosystems^2,3^. These marine environments accommodate enormous biodiversity from biological, ecological, physical, and chemical perspectives due to the specificity of habitats^4^. Over a long evolutionary process, biological interactions have shaped innumerable organisms to develop unique adaptation mechanisms amidst fierce competition for survival^5^. Marine bacteria, which have evolved under diverse and sometimes extreme oceanic conditions, represent a promising yet underexplored reservoir of genetic elements that could address current limitations in synthetic biology toolkits^6^.

Despite the great variety of organisms adapted to specific conditions in marine environments, reports of unique genetic resources from these ecosystems remain relatively rare. This can be attributed to the fact that the marine microbial community, which possesses the most extensive biodiversity and unique metabolic, functional, and structural characteristics, has not been sufficiently studied. Although more than 1.2 million species have been taxonomically classified thus far, approximately 91% of currently existing marine species remain unknown^7^. Over the past few decades, researchers have shown renewed interest in identifying marine microbes and harnessing these useful resources either for biodiversity research or for their economic significance, resulting in the discovery of several marine chassis microorganisms containing unique genetic resources adapted to extreme environments^8–10^.

*Vibrio* species represent highly adaptable Gram-negative bacteria with remarkable genetic plasticity, enabling their survival across diverse marine and brackish environments^11^. Their rapid growth rates (generation times of < 10 min), sophisticated quorum sensing systems, and ability to persist in both free-living and host-associated states make them particularly valuable as potential chassis organisms in synthetic biology^12–16^. Unlike many other bacterial models, *Vibrios* have evolved unique mechanisms to maintain cellular homeostasis under extreme pH conditions, particularly in alkaline environments commonly encountered in marine settings or during host colonization, where ammonia production can elevate pH levels^17^. This adaptability is achieved through coordinated expression of specific stress-response genes regulated by environmentally-responsive promoters. Of particular interest are proteins involved in maintaining protein folding integrity under stress conditions, such as PPIases, which serve as molecular chaperones to prevent protein aggregation and maintain cellular function^18–20^.

To date, we have attempted to expand our understanding of *Vibrio* survival under extreme environmental stress through genetic and proteomic analyses of *V*. *anguillarum*^18–23^. In this study, we report the first identification and characterization of promoter regions in *V*. *anguillarum* NB10 that drive increased expression of VaFklB under extremely alkaline conditions. Understanding the regulatory elements controlling these stress-responsive genes provides not only fundamental insights into bacterial adaptation but also practical genetic tools for engineered biological systems with applications in biotechnology, biomanufacturing, and environmental remediation.

## Results

### Proteomic identification of VaFklB as dramatically upregulated under extreme alkaline stress

**Fig. 1:**
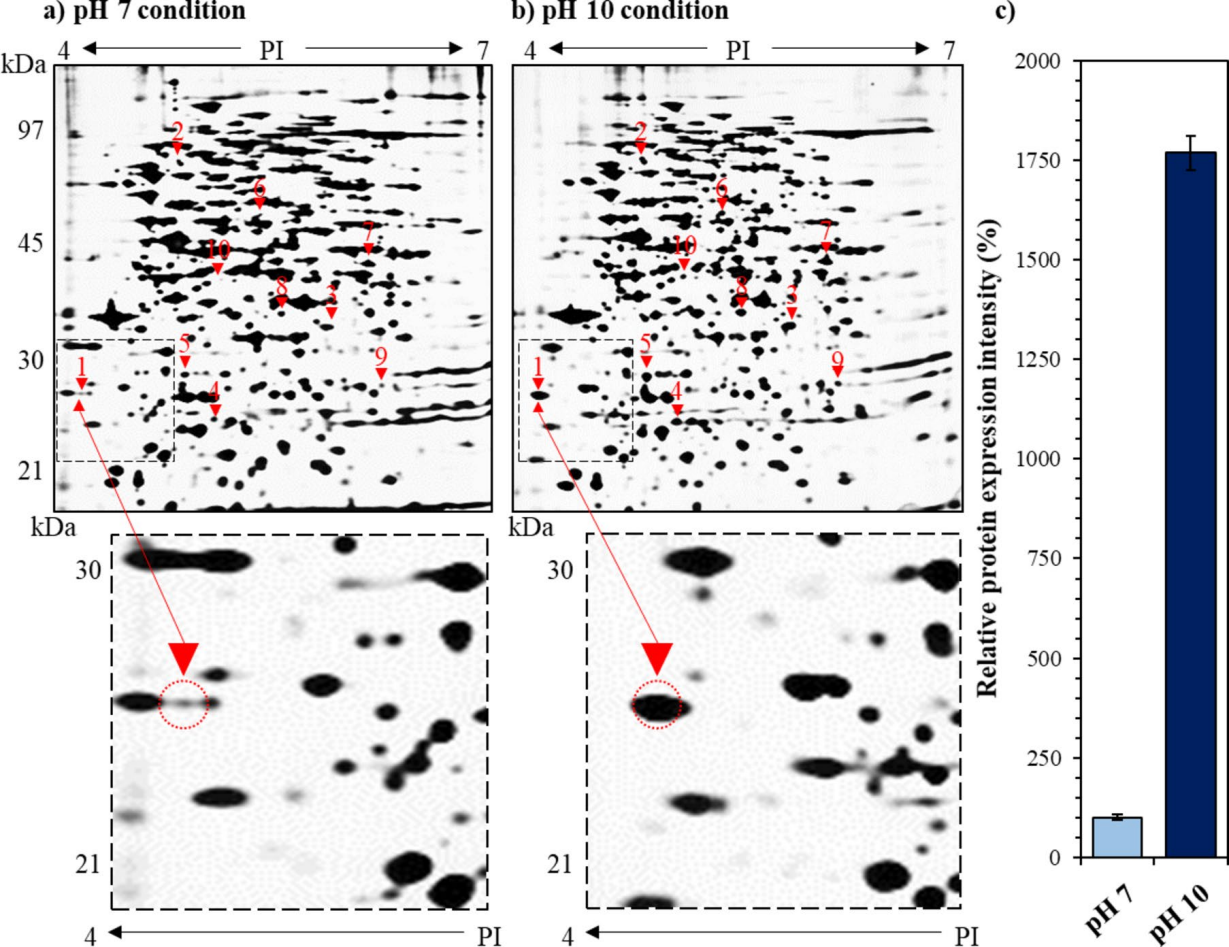
Comparative proteomic analysis of NB10 under pH 7 and pH10 alkaline stress conditions. (**a**) Non-stress condition; 25 °C and pH 7, (**b**) pH stress condition; 25 °C and pH 10, and (**c**) the expression differences of VaFklB in each environment. The gel images labeled (**a**,** b**) represent a single image from three independent experiments. Each spot indicates protein expression, with the 10 selected proteins that are upregulated at pH 10 marked by red triangles and spot numbers. The spot numbers correspond to those listed in Table 1. The gel images were manually adjusted for brightness and contrast to enhance the differentiation between protein spots. The original gel images and the images from the adjustment stages are provided in the supplementary material (Fig. S1).

Proteomic analysis of *V. anguillarum* NB10 (serotype O1) using two-dimensional (2D) sodium dodecyl sulfate-polyacrylamide gel electrophoresis (SDS–PAGE) (2-DE) revealed that a total of 10 proteins were significantly upregulated under extreme alkaline stress. Ultimately, the comparative analysis based on MALDI-TOF MS/MS and NB10 genome (accession number: PRJEB5701) identified five of these upregulated proteins: peptidyl-prolyl cis/trans isomerase (FklB; Protein ID; CDQ51236.1), general secretion pathway protein D (CDQ49073.1), cysteine synthase A (CDQ49756.1), deoxyribose-phosphate aldolase (CDQ51041.1), and transcriptional regulator OmpR (CDQ49100.1) (Table 1). Particularly, protein spot density-based expression analysis showed that the peptidyl-prolyl cis/trans isomerase (VaFklB) showed a 1,768.1 ± 43.2% higher expression level under extreme alkaline stress condition (25 °C and pH 10) compared to the normal condition (25 °C and pH 7) (Fig. 1). Therefore, in silico analysis was conducted to characterize VaFklB, which exhibited the highest upregulated expression level at pH 10 among the five proteins, along with its potential as an alkaline-inducible promoter.

**Table 1:** Profiles of up-regulated proteins in *V*. *anguillarum* NB10 against pH 10 stress condition.

Spot no.	Protein name	Locus (Chr. 1)	Protein ID	MW (kDa)	Molecular function	Score MASCOT
1	Peptidyl-prolyl cis–trans isomerase	2,695,469–2,696,089	CDQ51236.1	29	Peptidyl-prolyl cis–trans isomerase	164
2	General secretion pathway protein D	185,777–187,810	CDQ49073.1	73	Outer membrane secretin component of the type II secretion system involved in transporting exoproteins from the periplasm	141
3	Cysteine synthase A	990,531–991,499	CDQ49756.1	34	Catalyzes the last reaction of L-cysteine synthesis in bacteria	115
4	Deoxyribose-phosphate aldolase	2,464,018–2,464,794	CDQ51041.1	28	The conversion of exogenous deoxyribonucleosides for energy generation	194
5	Transcriptional regulator OmpR	213,902–214,621	CDQ49100.1	28	Member of the two-component regulatory system envZ/ompR involved in the regulation of osmoregulation	76
6	6-phospho-β-glucosidase	–	–	21	Hydrolase; 6-phospho-beta-glucosidase	58
7	Glucose-1-phosphate adenylyl-transferase	–	–	45	Glucose-1-phosphate adenylyl transferase; transferase; nucleotidyl transferase	68
8	Fructose-1-phosphate kinase	–	–	35	1-Phosphofructokinase; phosphotransferase	46
9	Threonine dehydratase	–	–	30	l-Threonine ammonia-lyase	62
10	Hypothetical	–	–	44	–	65

### In silico characterization of alkaline-inducible VaFklB

**Fig. 2:**
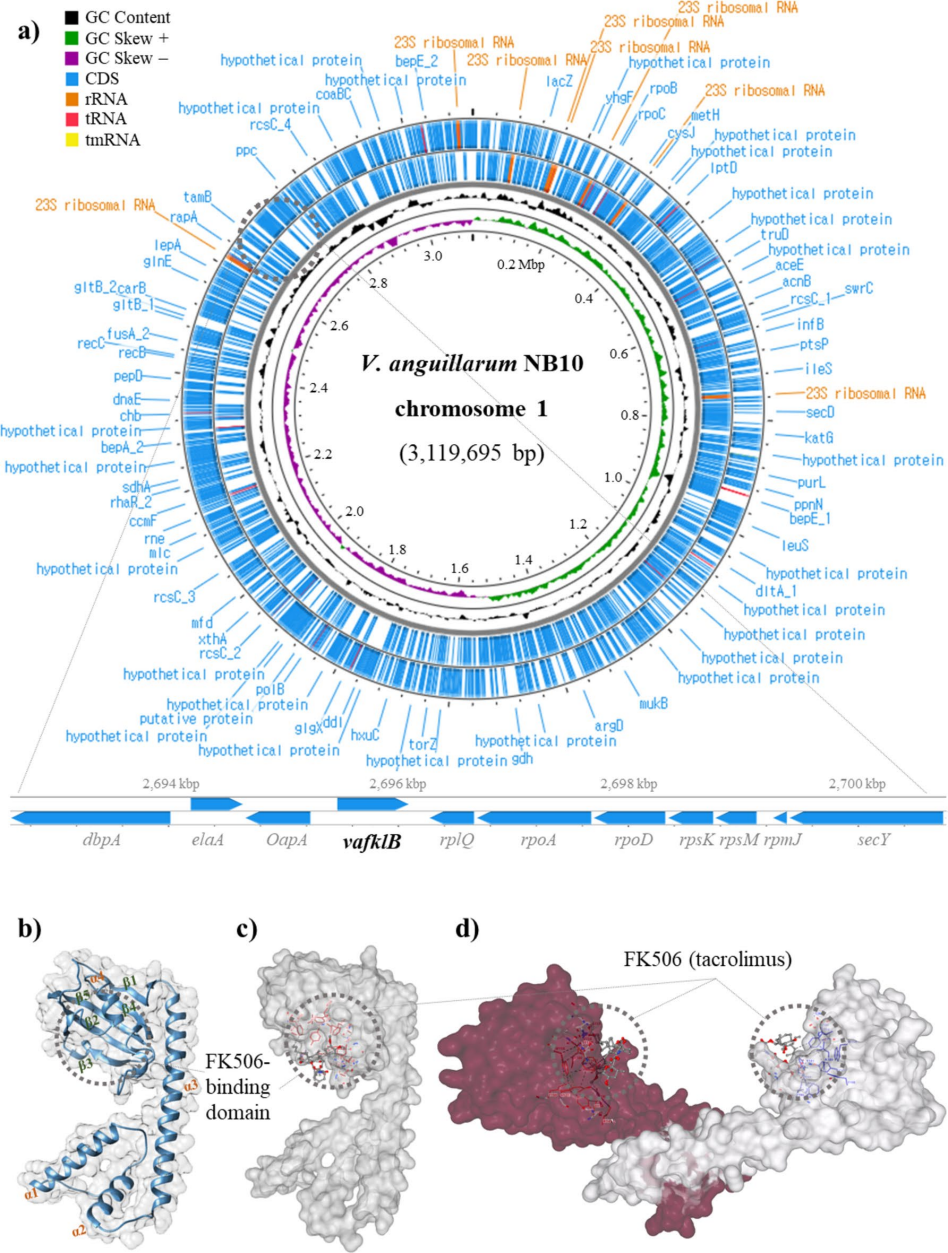
The *vafklB* locus within chromosome 1 of NB10 and the predicted protein structures of VaFklB bound to FK506 (tacrolimus). The protein structures and ligand docking models are based on bioinformatics predictions. (**a**) Circular plot of the genome of chromosome I and the *vafklB* gene locus. (**b**) Monomer topology of VaFklB represented as a helix and strand. (**c**) Schematic representation of the predicted protein structure of the VaFklB monomer with a surface view bound to FK506. (**d**) The VaFklB homodimer complex bound to FK506.

Figure 2 shows the location, three-dimensional structure, and ligand-binding site of VaFklB and its adjacent genes on the NB10 chromosome I (chr. 1). According to genome analysis, NB10 is a multi-chromosome bacterium harboring major chromosomes (3.12 Mbp), extra-chromosomes (chr. 2; 1.12 Mbp), and plasmid (67 Kbp). The *vafklB* gene that is up-regulated under extreme alkaline stress was located on chr 1 (gene location; 2,695,469–2,696,089) (Fig. 2a). The *vafklB* gene encodes a type of FKBP and is present as a single gene rather than as an operon. It is located adjacent to *oapA* (opacity associated protein A), *elaA* (putative acetyltransferase), and *dbpA* (putative ATP-dependent RNA helicase) in the 5’-upstream direction and *rplQ* (50 S ribosomal protein L17), *rpoA* (RNA polymerase alpha-subunit), and *rpsD* (30 S ribosomal protein S4) genes in the 3’-downstream direction. VaFklB comprises conserved amino acids, namely, Tyr^127^, Gly^129^, Phe^137^, Asp^138^, Val^154^, Ile^155^, Trp^158^, and Tyr^181^ in the C-terminal hydrophobic cavity as part of a FK506 binding pocket, which is a 23-membered macrolide lactone tacrolimus known for its immunosuppressive activity. The putative structure comprises four α-helices and five β-strands in the form of a β-sheet with a 1–4–5–2–3 topology wrapped around helix α4 (Fig. 2b). Molecular docking analysis showed that tacrolimus was bound to the C-terminal binding pocket, suggesting that VaFklB is a type of FKBP (Fig. 2c). Additionally, it is a homodimer characterized by an N-terminal α-helical structure known for its dimerization domain through hydrophobic interactions, as previously reported^19,20,24,25^, suggesting the possibility of a periplasm-localized protein in NB10 (Fig. 2d).

### Analysis of putative promoter regions expressed under alkaline conditions

**Fig. 3:**
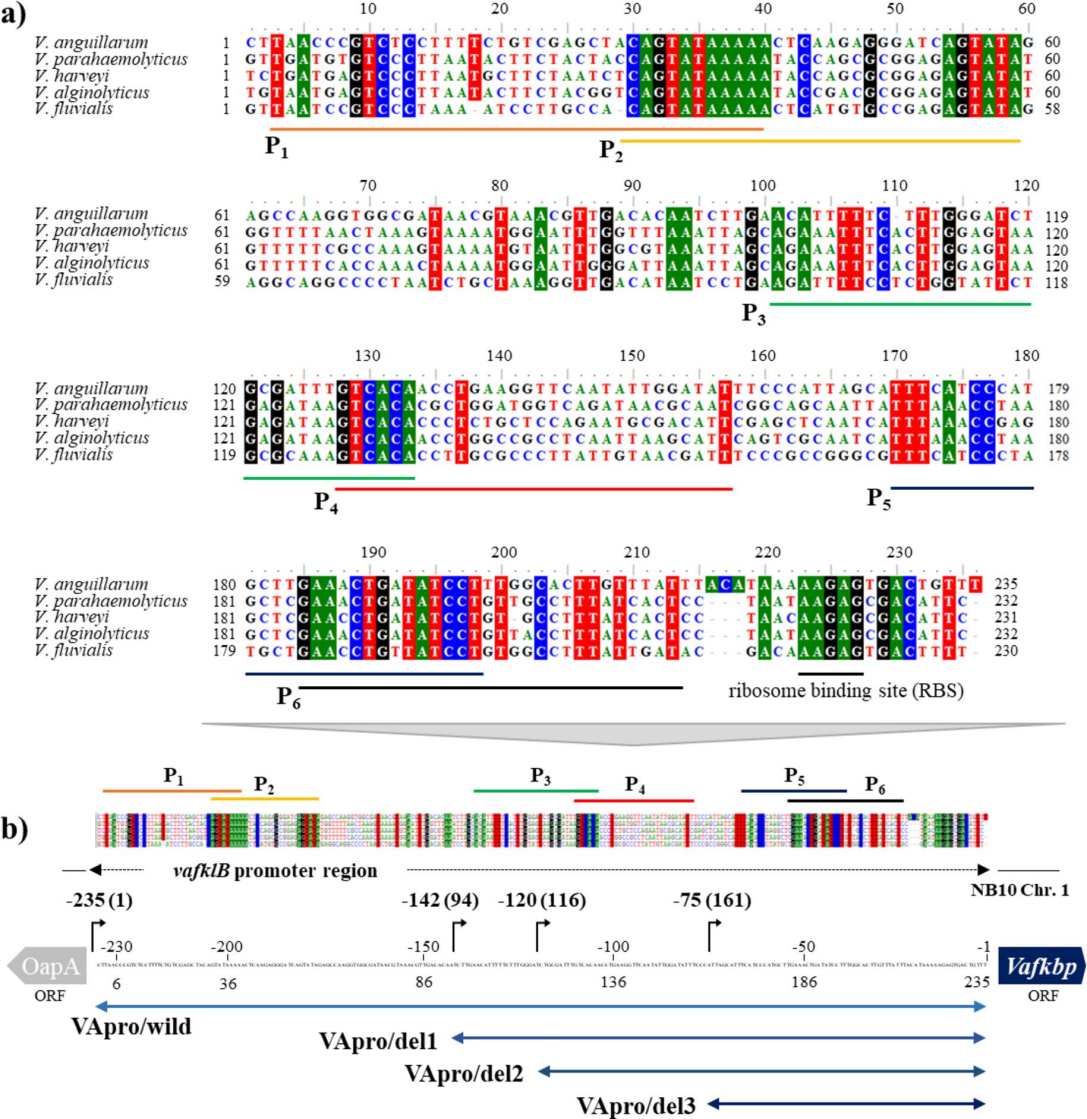
Conserved regions of the 5ʹ untranslated region of *vafklB*, and selected predicted promoter regions. (**a**) Comparative sequence analysis of the untranslated region from *V. anguillarum* NB10 in the 5ʹ direction of *vafklB* with the untranslated regions corresponding to *vafklB* from related species of the genus *Vibrio*. (**b**) Design of stepwise deleted recombinant promoter regions to identify the core promoter regions. The color shading represents the matching nucleotides among the different *Vibrio* species. P1 to P6 represent the selected promoter regions.

VaFklB exhibited amino acid sequence similarities of 91.7%, 92.2%, 87.8%, and 89.8% with the peptidylprolyl isomerases (protein ID: AMG05761.1) from *V*. *parahaemolyticus* ATCC 17,802, peptidylprolyl isomerase (AIV04382.1) from *V*. *harveyi* ATCC 33,843, FKBP-type 22 kDa peptidyl-prolyl cis-trans isomerase (AGV18288.1) from *V*. *alginolyticus* ATCC 17,749, and the FKBP-type 22 kDa peptidyl-prolyl cis-trans isomerase (WDY52166.1) from *V*. *fluvialis* 10 M-VF, respectively. Additionally, the nucleotide identity of the upstream untranslated sequence containing the potential promoter regions of *vafklB* revealed similarities of 52.9%, 50.8%, 50.8%, and 59.7% with *V*. *parahaemolyticus* ATCC 17,802, *V*. *harveyi* ATCC 33,843, *V*. *alginolyticus* ATCC 17,749, and *V*. *fluvialis* 10 M-VF, respectively. In particular, nucleotide sequence homology at the 30–40, 54–59, 129–133, 170–177, 185–200, and 220–231 bp regions was found to be significantly consistent between the sequence upstream of *vafklB* and the upstream region of the corresponding FKBP-type PPIase in species related to *V. anguillarum* NB10 (Fig. 3a). Therefore, these regions were arbitrarily designated as the -10 (Pribnow box) or -35 regions of the promoter sequence. Subsequently, six putative promoters (P1–P6) were selected such that each sigma factor-binding region was included in an arbitrary promoter sequence (Fig. 3b). Based on this, each partial sequence with the 5ʹ end deleted was identified and named as VApro/del1, VApro/del2, and VApro/del3, respectively, to identify the core promoter region expressed under alkaline stress. Table 2 lists the sequences of each putative promoter region.

**Table 2:** The sequence of each putative promoter region.

Promoter	Locus in	Putative promoter region (5’→3’)		
	Chr. I (bp)	-35	~ 16–18 bp	-10
P_1_	2,695,242–2,695,271	GTCTCC	TTTTCTGTCGAGCTACAG	TATAAA
P_2_	2,695,366–2,695,395	CAGTATA	AAAACTCAAGAGGGATC	AGTATA
P_3_	2,695,322–2,695,351	GAACAT	TTTTCTTTGGGATCTGCG	ATTTGT
P_4_	2,695,350–2,695,379	GTCACA	ACCTGAAGGTTCAATATT	GGATAT
P_5_	2,695,392–2,695,419	TTTCAT	CCCATGCTTGAAACTG	ATATCC
P_6_	2,695,408–2,695,437	AAACTG	ATATCCTTTGGCACTTGT	TTATTT

### Analysis of the potential alkaline-inducible promoter region in *E*. *coli* system

**Fig. 4:**
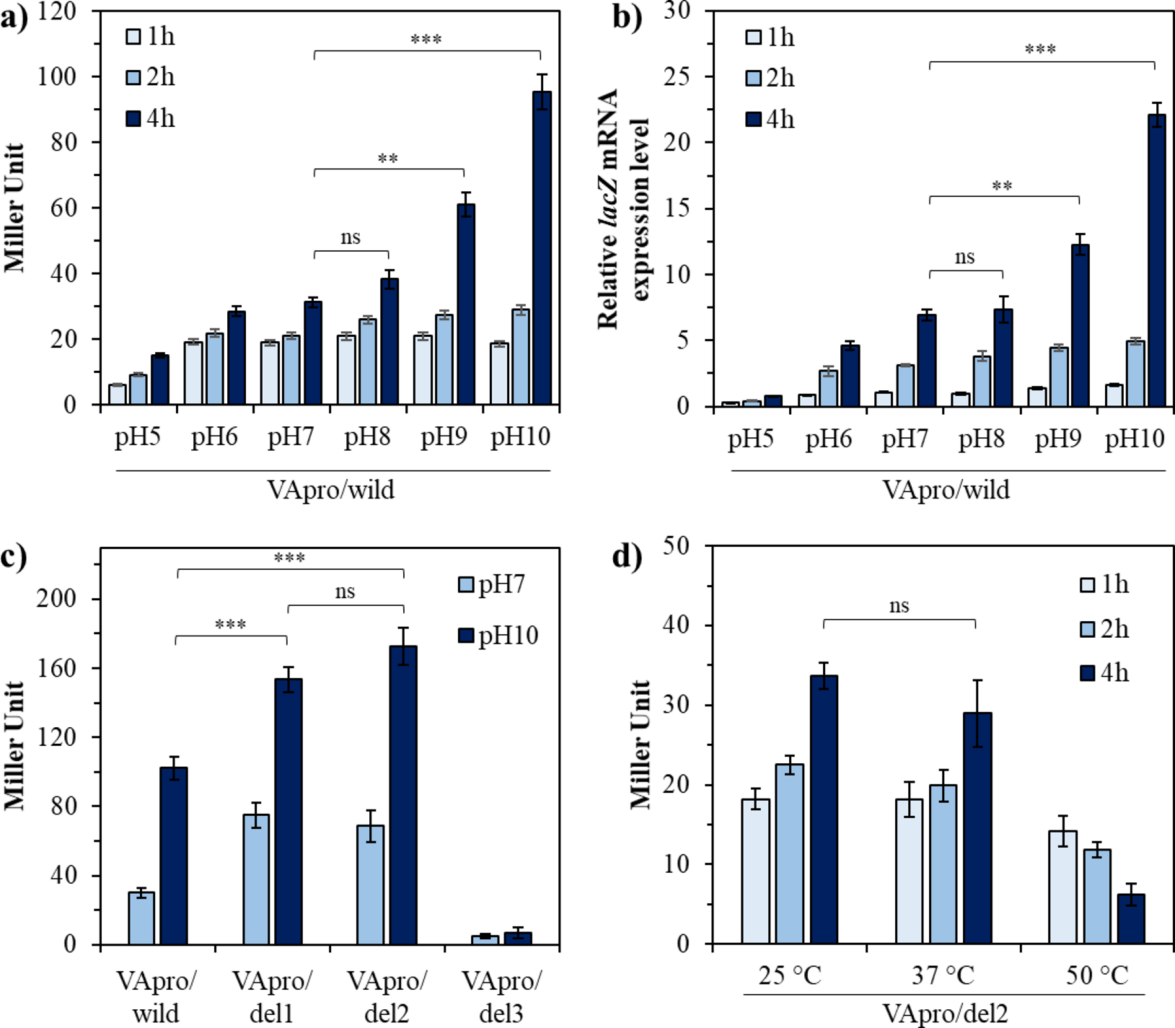
Promoter strengths of selected potential *vafklB* promoters at different pH and temperature in the *E*. *coli* system. (**a**) Promoter strength of VApro/wild converted to β-galactosidase activity. (**b**) Promoter strength of VApro/wild measured based on lacZ mRNA expression. (**c**) Promoter strength of stepwise deleted promoters VApro/del1, VApro/del2, and VApro/del3 with VApro/wild at different pH and time durations, and (**d**) the expression level of VApro/del2 after heat shock in an *E*. *coli* system.

The potential alkaline-inducible promoter that was upregulated at high pH in *V. anguillarum* NB10 was also effectively implemented in the *E. coli* system. Significant promoter expression was confirmed in the recombinant *E. coli* pVApro/wild-type after 4 h under pH 9 and 10 stress (Fig. 4a). Furthermore, promoter expression increased over time under all tested pH conditions with the highest expression rate observed at 4 h. β-galactosidase activity was 61.47 ± 2.91 and 95.83 ± 6.76 MU, respectively, at pH 9 and 10 after 4 h of stress, which was 1.97- and 2.88-fold higher than that observed normal conditions of 25 °C and pH 7 (31.27 ± 1.15 MU). Additionally, the *lacZ* mRNA expression rate showed a pattern similar to that of the enzyme activity observed earlier in Miller units. The mRNA expression level was 6.94 ± 0.39 after 4 h at pH 7, and the expression levels increased to 12.27 ± 0.77 and 22.12 ± 0.9 at pH 9 and 10, respectively (Fig. 4b). In contrast, β-galactosidase activity and mRNA expression at pH 8 were 38.43 ± 2.46 MU and 7.34 ± 0.99, respectively, which were higher on average than those at pH 7; however, the difference was not significant.

The selective expression of the stepwise-deleted promoter was measured at different pH levels (Fig. 4c). β-galactosidase activity pertaining to VApro/del1 and VApro/del2 was 75.12 ± 7.3 MU and 68.6 ± 9.2 MU, respectively, at pH 7 and after 4 h, which was 2.5- and 2.3-fold higher compared with the 30 ± 2.8 MU detected for VApro/wild. Similarly, the activities were increased 1.5- and 1.6 times, reaching 153.4 ± 7.4 and 172.6 ± 12.3 MU, respectively, compared to the 102.1 ± 6.8 MU detected for VApro/wild at pH 10. The promoter strength of VApro/del2 was higher on average than that of VApro/del1 at pH 10; however, the expression levels of VApro/del1 and VApro/del2 did not differ significantly. VApro/del3 exhibited significantly lower promoter strength of 4.8 ± 1.2 and 6.8 ± 3.2 MU at pH 7 and pH 10, respectively. Meanwhile, no significant increase in the expression of VApro/del2 was observed during heat shock at 37 °C and 50 °C compared to 25 °C in the *E*. *coli* system (Fig. 4d).

### The *VafklB* gene expression in NB10 under stress conditions

**Fig. 5:**
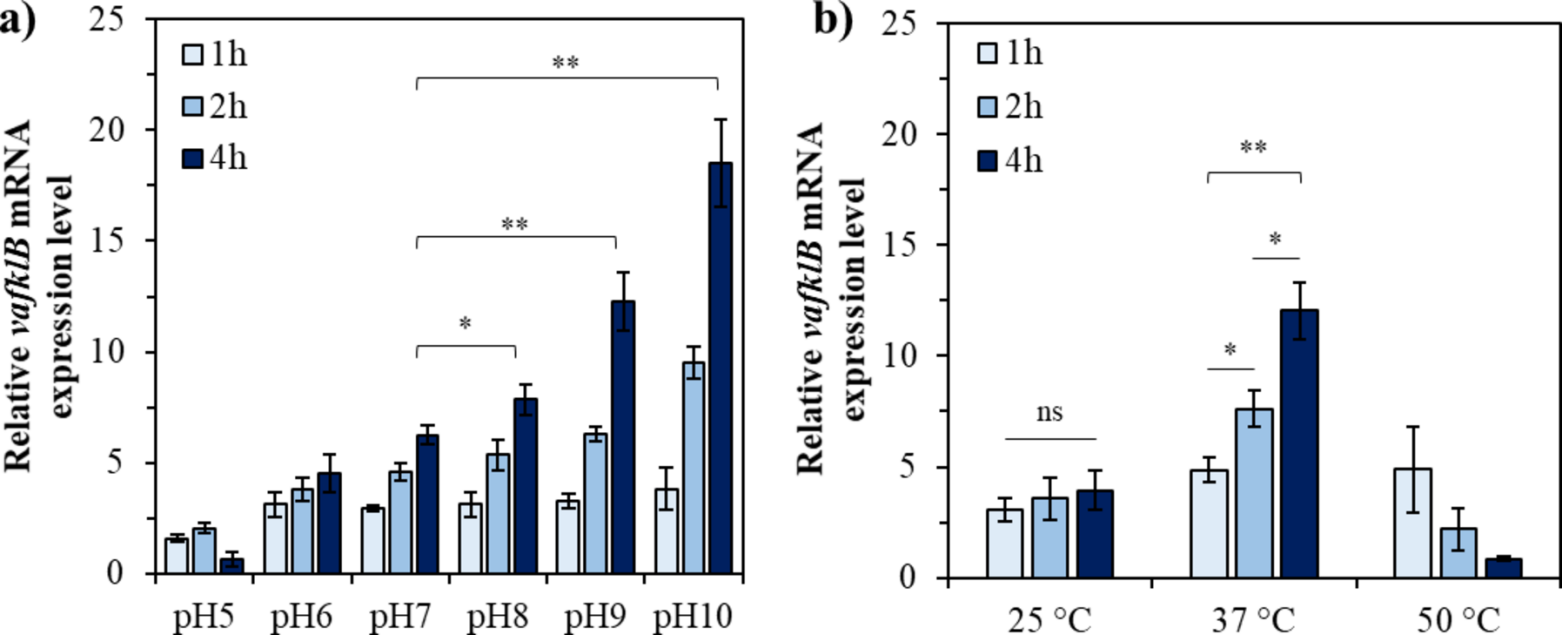
The *vafklB* gene expression in NB10 under stress conditions. Relative expression levels of *vafklB* under (**a**) various pH conditions and (**b**) temperature stress.

The upregulation of *vafklB* under stress conditions identified in the proteomic analysis was validated at the gene level (Fig. 5). The expression of *vafklB* in NB10 at each pH was found to be very similar to the expression pattern observed by the *E*. *coli* system with VApro/wild. The temporal expression of *vafklB* in alkaline environments from pH 8 to 10 significantly increased, with relative expression rates measured at 4 h post-treatment being 7.85 ± 0.68, 12.26 ± 1.33, and 18.49 ± 1.95 for pH 8, 9, and 10, respectively. The expression rates after 4 h in the stress environment were approximately 1.26, 1.9, and 3.0 times higher at pH 8, 9, and 10 compared to pH 7. Meanwhile, for *Vibrio*, which has an optimal growth temperature of 25–28 °C, the relative expression rates of *vafklB* under high-temperature stress at two temperatures were 4.87 ± 0.52, 7.63 ± 0.85, and 12.03 ± 1.28 at 37 °C for 1 h, 2 h, and 4 h, respectively, while at 50 °C, downregulation was observed.

## Discussion

In this study, five out of the 10 proteins that were significantly upregulated in NB10 under extreme alkaline stress were identified as the peptidyl-prolyl cis–trans isomerase VaFklB, general secretion pathway protein D, cysteine synthase A, deoxyribose-phosphate aldolase, and transcriptional regulator OmpR. Moreover, the potential alkaline-induced promoter region of VaFklB, which exhibited the highest level of upregulation, was identified for the first time through genome and reporter gene expression analyses. The upregulation of these proteins in NB10 under extreme alkaline conditions is primarily associated with various physiological and metabolic changes that enable the cells to survive and adapt. The probability of evolving unique biological systems in response to geographically localized factors such as pH, temperature, salinity, dissolved oxygen, and nutrient requirements is high in marine microorganisms^26^. Particularly, fish-related pathogenic microorganisms have developed specific defense systems to survive against various environmental changes upon penetrating host bodies. One of these mechanisms is the expression of chaperone genes to ensure the stability of protein function under alkaline conditions such as those created by the production of ammonia and amines during fish decomposition.

Bacterial PPIases function as chaperones that play a critical role in protein folding and the regulation of cellular functions^27^. PPIases are classified into three types: FKBPs, which bind FK506; cyclophilins, which bind cyclosporin A; and parvulins, which are structurally distinct. In this study, VaFklB identified from NB10 possessed the dimerization domain of the helix-loop-helix (HLH) motif at the N-terminus, characteristic of typical FKBPs, as well as the FK506 binding domain at the C-terminus, which is composed of β-sheets and played a crucial role in protein refolding^19,20^. Furthermore, in silico analysis predicted that VaFklB contained conserved residues that play a significant role in promoting FK506 binding, with FK506 expected to bind precisely within the hydrophobic pocket formed by these residues at the C-terminus. As chaperones, FKBPs interact with the exposed hydrophobic surfaces of misfolded intermediates through this hydrophobic pocket, converting the misfolded proteins to their native-like forms and restoring enzymatic activity^19,28^. One of our major hypotheses regarding the upregulation of VaFklB is that it acts as a protein refolding-related chaperone, functioning to recover protein misfolding under extreme stress conditions. Therefore, the promoter that increases VaFklB expression in an alkaline environment is most certainly a part of an evolved system for the survival of *Vibrio* in response to changes in the host. Several studies have reported that FKBP type PPIases in marine-related Gram-negative bacteria are involved in protein refolding as molecular chaperones under various environmental stresses. For example, SIB1 FKBP22 from *Shewanella* sp. binds to a folding intermediate protein SIB1; PaFkbA from *Pseudomonas aeruginosa* is a periplasmic chaperone for protein folding; and VaFKBP22 and VaFKBP17 from *V. anguillarum* act as chaperones and co-chaperones to prevent thermal aggregation^19–21,24,29,30^. These previous studies also support the implication that the alkaline-inducible promoter for VaFklB expression assists protein refolding under the particular stress environment of *V. anguillarum* NB10. The general secretion pathway protein D plays a crucial role in transporting proteins into the periplasm or into the extracellular medium^31^. Its increased expression under alkaline conditions is thought to facilitate the secretion of stress-related proteins or enzymes, thereby aiding in environmental adaptation. Cysteine synthase A is an enzyme responsible for synthesizing cysteine, a precursor of the antioxidant glutathione^32^. Its expression may increase in response to elevated oxidative stress induced by reactive nitrogen species in extreme alkaline environments. Deoxyribose-phosphate aldolase, which is classified as a ‘survival gene’, is involved in carbohydrate metabolism, particularly in supplying precursors necessary for DNA repair and synthesis^33^. The expression of this enzyme could be upregulated to promote DNA repair under alkaline stress conditions^34^. Transcriptional regulator OmpR is a transcription factor that regulates responses to environmental stress, primarily by modulating membrane permeability^35^. In high alkaline environments, the expression of OmpR may increase to maintain membrane stability and assist in environmental adaptation. Additionally, while precise annotations are lacking, the upregulated proteins presumed to be 6-phospho-β-glucosidase, glucose-1-phosphate adenylyltransferase, fructose-1-phosphate kinase, and threonine dehydratase are also related to carbohydrate metabolism, energy storage, and amino acid metabolism. Their increased expression in extreme alkaline environment may indicate a role in maintaining cellular energy metabolism^36,37^.

Our findings demonstrate that the potential alkaline-inducible promoter of *vafklB* can be activated under extremely alkaline conditions in the *E*. *coli* system. However, the precise compatibility mechanism by which the foreign *Vibrio* promoter activates in *E*. *coli* remains ambiguous. Although many bacteria are vulnerable to harsh pH fluctuations, their defense mechanisms against pH stress remain unclear. Moreover, acid resistance mechanisms are well known but the alkaline resistance mechanisms remain largely uncharacterized. Nevertheless, several mechanisms that enable *E*. *coli* to sense external pH fluctuations and convert this information into internal signals for regulating the transcription of specific genes have been identified. The periplasmic protein YceI (multidrug exporter), outer membrane porin proteins OmpC (outer membrane porin C) and OmpA (outer membrane protein A), and membrane-bound redox regulator DsbA (periplasmic dithiol oxidoreductase) have been induced in *E. coli* to respond to osmotic pressure under extremely high alkaline conditions^36–38^. As part of a strategy to produce organic acids to maintain cytosolic pH homeostasis, alkaline pH was used to stimulate the amino acid metabolic enzyme tryptophanase (TnaA) to produce NH_3_ and acids^39^. Moreover, AstD (succinylglutamic acid semialdehyde dehydrogenase), GadA and GadB (glutamate decarboxylases), and GabT (γ-aminobutyric acid transaminase), which participate in the arginine and glutamate catabolic pathways, were also expressed at high levels under alkaline stress^37,39,40^. In the case of *V. cholera*, membrane-embedded transcriptional regulators and their respective partner proteins ToxRS and TcpPH activate toxT (cytoplasmic protein) expression, which encodes the master regulator for the transcription of the downstream genes *tcp* (toxin co-regulated pilus) and *ctx* (cholera toxin)^41^. Furthermore, ToxRS coordinates the inverse regulation of the outer membrane porins OmpU and OmpT to build resistance to alkali stress. Additionally, ompR-induction at alkaline pH results in the transcriptional silencing of the acid tolerance response (ATR) and virulence genes. Moreover, OmpR contributes to fitness at alkaline pH and activates the expression of chiP, which is a chitin-specific porin^17^. Furthermore, another survival strategy is to maintain the cytoplasmic pH at a much lower level than that of the highly alkaline external environment through the regulation of multiple resistance and pH adaptation (Mrp) multi-subunit cation/proton antiporters^42,43^. In summary, the two Gram-negative bacteria use two representative strategies to respond to alkaline stress: maintaining pH homeostasis by producing organic acids and metabolites in the cytoplasm and controlling osmotic pressure by regulating cell membrane proteins. Furthermore, the alkaline response strategies of Gram-negative bacteria such as *E. coli* and *Vibrio* appear to overlap with those against salt and extracellular envelope stress due to increased sodium cytotoxicity at high pH and sensitivity of certain cell wall synthesis enzymes at fluctuating pH. This suggests that the intermediate signaling mechanisms for transmitting signals to the promoter in recognition of alkaline conditions may be compatible between Gram-negative bacteria.

State-of-the-art genetic engineering technologies include various tools and methods for manipulating gene expression. However, these genetic tools must be expanded into practical tools that can be used to directly control the expression of genes selected for designing optimized metabolic pathways based on metabolic engineering, synthetic biology, and biofoundry. Among the various tools, promoter engineering has emerged as a powerful tool for redesigning the expression of gene clusters found in bacterial genomes. Generally, intracellular metabolic fluxes are regulated by a series of distinct but intertwined regulatory controls that occur at the transcriptional, translational, and protein levels. One of fundamental means to alter this metabolic flux is to control transcript production at the promoter level. Most of these methods rely on the regulation of transcription initiation using various promoters^44^. Therefore, designing metabolic pathways has long relied on effective promoter discovery and characterization. Furthermore, the field of promoter engineering attempts to modulate promoter transcriptional ability by mutating the promoter DNA sequence, and the identification and characterization of existing promoters represents a way forward^45^. The exploration of conserved regions in bacterial promoters is crucial for the identification and characterization of promoters. The conservation of specific nucleotide residues across different bacterial species suggests functional importance. One of the most prominent features of promoter sequences is the presence of recognition elements for the RNA polymerase (RNAP) holoenzyme (Eσ)^46^. Through the long history of comparing promoter sequences, it has been identified that the -10 element (Pribnow box) and the -35 region, which play critical roles in RNA polymerase binding and transcription initiation, are highly conserved in many bacterial promoters^47–52^. This conservation indicates that these elements are fundamental to bacterial gene regulation and have been maintained through evolutionary pressure. Studies have revealed that these promoter elements are well-conserved across various bacterial species. The regulatory elements of the *rpoH* gene promoter, including the -35 and -10 regions, demonstrated significant sequence similarity among enteric bacteria such as *E*. *coli*, *Salmonella enterica*, and *Shigella flexneri*^53^. This conservation underscores the importance of these regions in transcriptional regulation. The 5ʹ untranslated region of bacteria influences both transcription and translation, playing a crucial role in regulating gene expression. Multiple promoters can exist within the 5ʹ untranslated region, allowing specific promoters to be activated only under certain environmental conditions during various stress situations, thereby enabling more refined control of gene expression^54–56^. This multiple promoter system allows bacteria to express necessary proteins in appropriate amounts at the right times, enhancing the flexibility of gene expression and maximizing survival and adaptability. FKBPs not only function as chaperones but also influence various physiological processes such as bacterial pathogenicity, immune evasion, cell cycle regulation, and signal transduction^57^. The potential existence of multiple promoter in this functional context could be a key reason for the differential induction of various segments in the upstream region of *vafklB*. Furthermore, while a significant upregulation of VApro/del2 was not observed in *E*. *coli* under high-temperature stress at 50 °C (Fig. 4d), a temporal upregulation of *vafklB* was observed in NB10 under high-temperature stress at 37 °C, where the optimal growth temperature is 25–28 °C (Fig. 5b). This suggests the presence of at least two or more promoters within the 235 bp of the *vafklB* 5ʹ untranslated region, indicating the possibility of differential induction of these segments.

In this study, we identified highly conserved regions in the potential promoter area of the *vafklB* gene across different *Vibrio* species. Based on this finding, the potential core region of the alkaline-inducible promoter has been delineated. Although the exact transcription start site in the upstream region of *vafklB* was not mapped, the predicted region of the alkali-inducible promoter derived from our study can be effectively utilized to selectively express genes required in specific environments. Moreover, this promoter region is highly accessible for direct application in synthetic biology due to its compatibility with *V*. *anguillarum* and *E*. *coli*. We believe that the results obtained from optimizing and validating the alkaline-inducible promoter region could serve as a foundational tool for generating synthetic elements with desirable functions. The optimized *vafklB* promoter (VApro/del2) exhibited robust activity with absolute expression levels of 153.4 ± 7.4 and 172.6 ± 12.3 MU at pH 9 and pH 10, respectively, demonstrating significantly enhanced strength compared to the wild type. However, these values are relatively modest when compared to the strongest known expression systems in *E*. *coli*, such as the T7 promoter (13,000 MU) and Lac promoter (3800 MU), which operate in the presence of inducers like isopropyl β-D-1-thiogalactopyranoside (IPTG) or lactose^58,59^. Nevertheless, alkaline-inducible promoters offer distinct advantages, including precise expression control for toxic gene products or proteins that may negatively impact cell growth, and cost-effective regulation through simple pH adjustment, eliminating the need for expensive chemical inducers. The identification of the potential core region of the alkaline-inducible promoter, which is selectively expressed in extreme alkaline environments, represents the most significant finding of this study and will provide further insights into functional promoters that can be employed in biotechnological applications. Despite these findings, significant knowledge gaps remain regarding the precise molecular interactions that enable this cross-species promoter functionality. Future studies employing ChIP-seq analysis to precisely map RNA polymerase binding sites, identifying specific transcription factors activated under alkaline conditions, and determining the exact transcription initiation sites will greatly enhance our understanding of the molecular mechanisms that allow *vafklB* promoters to function effectively in diverse host systems, including *E*. *coli*.

In conclusion, we leveraged the power of proteomics and genome mining as biophysical tools to discover a logical and regulatory alkaline-inducible promoter that controls bacterial decision making. To the best of our knowledge, this is the first study to report a strong alkaline-inducible promoter. The characterization of novel promoters can help generate the dynamic range required to fine-tune gene expression for metabolic engineering applications. Therefore, this result is significant as it presents another promising and powerful gene regulation tool to control synthetic elements with desirable functions in synthetic biology applications.

## Methods

### Bacterial strains, culture conditions, and plasmids

*V. anguillarum* strain NB10 (serotype O1) was standard cultured aerobically under conditions of 125 rpm and 25 °C in brain heart infusion (BHI) media (Becton Dickinson and Co., Sparks, MD, USA)^60^. The *E*. *coli* strain DH5α and recombinant *E*. *coli* derived from DH5α, used for gene cloning and measuring promoter strength, were cultured under standard condition of 150 rpm and 37 °C in Luria-Bertani (LB) broth. All bacterial strains were stocked at − 80 °C in 25% glycerol and 7% dimethyl sulfoxide (DMSO) until used later. The pET28a(+) vector (Novagen, Madison, WI, USA) containing the T7 promoter and kanamycin resistance gene was used for cloning and expression. The pcDNA3.1/His/lacZ vector (Invitrogen, Life Technologies, Carlsbad, CA, USA) was used as the template for *LacZ* gene. The bacterial strains and plasmids used in this study are listed in Table 3.

**Table 3:** Bacterial strains and plasmids used in this work.

Strains or plasmids	Relevant properties	Reference or sources
Strains		
*V. anguillarum* NB10	Serotype O1	^60^
*E. coli* DH5α	F^−^ *end*A1 *rec*A1 Φ80d*lacZ*ΔM15; cloning strain	Laboratory collection
*E. coli* VApro/wild	*E. coli* DH5α containing pVApro/wild plasmid; Host for measuring promoter strength	This work
*E. coli* VApro/del1	*E. coli* DH5α containing pVApro/del1 plasmid; Host for measuring promoter strength	This work
*E. coli* VApro/del2	*E. coli* DH5α containing pVApro/del2 plasmid; Host for measuring promoter strength	This work
*E. coli* VApro/del3	*E. coli* DH5α containing pVApro/del3 plasmid; Host for measuring promoter strength	This work
Plasmids		
pET-28a(+)	a 5369 bp *E*. *coli* expression vector containing T7 promoter, F1 origin, and pBR322 origin	Novagen co.
pcDNA3.1/His/*lac*Z	a 8592 bp mammalian expression vector containing the *lac*Z gene for β–galactosidase	Invitrogen co.
pVApro/wild	A recombinant vector for measuring promoter strength; replaced the T7 promoter of pET-28a(+) with the 235 bp fragment of the FklB promoter region; harboring the lacZ gene derived from pcDNA3.1/His/lacZ as the reporter gene	This work
pVApro/del1	A recombinant vector for measuring promoter strength; replaced the T7 promoter of pET-28a(+) with the 142 bp fragment of the FklB promoter region; harboring the lacZ gene derived from pcDNA3.1/His/lacZ as the reporter gene	This work
pVApro/del2	A recombinant vector for measuring promoter strength; replaced the T7 promoter of pET-28a(+) with the 120 bp fragment of the FklB promoter region; harboring the lacZ gene derived from pcDNA3.1/His/lacZ as the reporter gene	This work
pVApro/del3	A recombinant vector for measuring promoter strength; replaced the T7 promoter of pET-28a(+) with the 75 bp fragment of the FklB promoter region; harboring the lacZ gene derived from pcDNA3.1/His/lacZ as the reporter gene	This work

### Differential expression proteome analysis based on environmental stress

#### Stress condition and total protein preparation

*V. anguillarum* NB10 was cultured in BHI broth at 25 °C and pH 7 until an optical density at 600 nm (OD_600_) of 0.6, corresponding to 5 × 10^8^ colony forming units per milliliter (CFU mL^−1^), was achieved. The cells were harvested by centrifugation at 12,000 rpm for 5 min, then resuspended and incubated in normal media (pH 7) or media conditioned under pH stress (pH 10) for 4 h at 25 °C with shaking at 150 rpm. *V. anguillarum* NB10 was harvested through centrifugation and vortexed thoroughly for 16 h in a lysis solution containing 7 M urea, 4% 3-(3-cholamidopropyl dimethylammonium) propane sulfonate (CHAPS), 2 M thiourea, 100 mM dithiothreitol (DTT), and 2% pharmalyte. After centrifugation, the supernatants were recovered and treated using a 2-D Clean-up Kit (GE Healthcare, Piscataway, NJ, USA). The purified total proteins were resuspended in the lysis solution. Protein concentrations were determined using a modified Bradford assay; bovine serum albumin was used as the standard^61^.

#### Two-dimensional polyacrylamide gel electrophoresis (2D-PAGE)

One-dimensional isoelectric focusing (IEF) and two-dimensional (2D) sodium dodecyl sulfate-polyacrylamide gel electrophoresis (SDS–PAGE) (2-DE) were performed according to the manufacturer’s protocol (GE Healthcare, Piscataway, NJ, USA)^62^. Briefly, immobilized pH gradient (IPG) Dry Strips (immobilized pH 4–7, 13 cm; GE Healthcare) were rehydrated for 16 h at 25 in the lysis solution. The purified protein samples (200 µg) containing a 0.5% IPG buffer (pH 4–7) and 5% bromophenol blue were applied to the rehydrated IPG Dry Strip using cups at the cathodic or anodic end of the strips. The strips were focused on Multiphor II electrophoresis units using a one-step linear voltage gradient increasing from 300 to 3,500 V in 1.5 h, the voltage was then kept constant at 3,500 V for an additional 20 h, giving a total of 2.9 kVh. The focused IPG strips were equilibrated for 20 min in a solution containing 50 mM Tris–HCl (pH 8.8), 6 M urea, 30% glycerol, 2% SDS, and 1% DTT. Then, the DTT in the solution was replaced with 0.5% iodoacetamide, and the process was continued for an additional 20 min. The equilibrated strips were applied to a 12% polyacrylamide gel (14 × 13 cm, 1.5 mm thick). Silver staining was performed to visualize the proteins according to the manufacturer’s protocol (Thermo Fisher Scientific, Cleveland, OH, USA). The intensity of the protein spots was quantified using the ImageJ program (RRID: SCR_003070).

#### Protein identification

Upregulated proteins were selected using silver staining. The selected spots were excised from the gels and transferred to an Eppendorf tube for in-gel trypsin digestion. Briefly, the excised gel spots were de-stained using a solution of acetonitrile and 50 mM NH_4_HCO_3_ mixed in a 1:1 ratio. The proteins were reduced in a solution containing 50 mM NH_4_HCO_3_ and 10 mM DTT for 1 h at 56 °C, followed by alkylation with a solution of 50 mM NH_4_HCO_3_ and 55 mM iodoacetamide for 1 h at 25 °C. Subsequently, the gel pieces were washed three times with 50 mM NH_4_HCO_3_ and dehydrated with acetonitrile. The dried gel pieces were digested with sequencing-grade trypsin (Promega, Madison, WI, USA) at 37 °C for 18 h. The resulting tryptic peptides were dissolved in 50% acetonitrile containing 0.5% trifluoroacetic acid and desalted using a ZipTip C18 pipette tip (Millipore, Billerica, MA, USA). The peptides were directly eluted onto matrix-assisted laser desorption ionization (MALDI) plates using a-cyano-4-hydroxy-cinnamic acid (CHCA) matrix solution (10 mg mL^−1^ CHCA in 0.5% TFA: 50% acetonitrile, 1:1). MALDI-time of flight/mass spectrometry (MALDI-TOF/MS) and MALDI-TOF/MS/MS were performed in reflection mode using a 4700 Proteomics Analyzer (Applied Biosystems, Foster City, CA, USA). The proteins were identified by searching the National Center for Biotechnology Information (NCBI) database using the MASCOT program (http://www.matrixscience.com/; RRID: SCR_014322).

### In silico method for identifying protein and promoter region

#### Genome analysis

The NB10 genome was analyzed using the complete genome sequence deposited at NCBI (accession number: PRJEB5701) to identify the VaFklB and its promoter region based on the results of the amino acid sequence confirmed using MALDI-TOF/MS. Genome analysis was performed according to a method reported previously with slight modifications^63^. Briefly, the NB10 genome annotations were performed using Proksee (https://proksee.ca/), Rapid Annotation using Subsystem Technology 2.0 (RAST; https://rast.nmpdr.org/), and Bacterial and Viral Bioinformatics Resource Center (BV-BRC; http://www.bv-brc.org/) services. The updated annotation results, based on the latest databases, were compared and analyzed alongside the results that had previously been annotated in NCBI to facilitate cross-validation in identifying the target proteins. The circular map was constructed using the BV-BRC circular viewer (https://www.bv-brc.org/view/Genome/).

#### Three-dimensional homology-modeling and ligand docking

Molecular modeling and docking process were performed according to the procedure described in the reported previously with slight modifications^64^. Briefly, for 3D structural modeling of the FklB, homologous proteins were screened using the AlphaFold v2 protein database (https://alphafold.ebi.ac.uk/) using an AI system that predicts protein 3D structure based on its amino acid sequence (AlphaFoldDB Identifier; A0A649YL14, *Vibrio* sp. THAF191d). Secondary screening was performed on the protein templates using the NCBI (https://www.ncbi.nlm.nih.gov/structure/) and RCSB (https://www.rcsb.org/) servers. The candidate templates were applied to a protein structure homology modeling server (Swiss-Model; https://swissmodel.expasy.org/) to select templates for the structures that were determined using X-ray crystallography. The final predicted tertiary structures were constructed based on the selected template (PDB ID; 7dek.2.A, *Pseudomonas aeruginosa* FK506-binding protein PaFkbA). The conserved amino acid residues of VaFklB were determined through a homology analysis with the amino acid sequences of previously reported FKBPs, human 12 kDa FKBP (hFKBP12)^65^, *V*. *anguillarum* 17 kDa FKBP (VaFKBP17)^20^, *E*. *coli* 22 kDa FKBP^66^, and the macrophage infectivity potentiator (MIP) protein from *Legionella pneumophila* (LpMIP)^67^ using the BioEdit 7.2 program (RRID: SCR_007361). The 3D conformers of FK506 (PubChem CID: 445643; C_44_H_69_NO_12_; Tacrolimus) that act as FKBP ligands were visualized using the PubChem Chemical Molecules Database (https://pubchem.ncbi.nlm.nih.gov/). The intermolecular binding sites and affinities of the proteins and ligands were predicted and evaluated using the CB-Dock2 server (https://cadd.labshare.cn/cb-dock2/). Visualization and further analysis of the molecular complexes for parameters such as density maps, trajectories, and structure matching were performed using UCSF ChimeraX (https://www.cgl.ucsf.edu/chimerax/; RRID: SCR_015872) program.

#### Sequence alignment of the promoter region

To gain insights into potential promoter regions, the homologous proteins corresponding to FklB of NB10 were traced in other *Vibrio* species based on the reported genomic sequences and annotation information (*V. parahaemolyticus* ATCC 17802, accession number CP014046.2; *V. harveyi* ATCC 33843, CP009467.2; *V. alginolyticus* ATCC 17749, CP006718.1; and *V. fluvialis* 10 M-VF, CP118599.1). Subsequently, the sequences of the entire untranslated region containing potential promoter regions located upstream of the 5ʹ primer direction of *vafklB* or its homologous genes were secured through comparative genome analysis. The amino acid sequence homology and nucleotide sequence identity were calculated using BioEdit 7.2.

### Molecular cloning and constructions of recombinant vector

pVApro (pET-28a(+)/*vafklB* promoter/lacZ) was constructed by inserting the *vafklB* promoter and *lacZ* gene into pET-28a(+) as the backbone vector. The 3.1 Kbp *lacZ* gene was amplified using PCR (TaKaRa, Kyoto, Japan) with pcDNA3.1/His/lacZ as the template and complementary primer sets containing BamHI and XhoI restriction enzyme sites for gene cloning (New England Biolabs (NEB), Cambridge, MA, USA). The PCR conditions were 25 amplification cycles of 97 °C for 30 s, 58 °C for 60 s, and 72 °C for 30 s. The amplified *lacZ* and pET-28a(+) were digested using BamHI and XhoI and inserted into the multiple cloning site (MCS) of pET-28a(+) using T4 DNA ligase (Takara, Kyoto, Japan). Then, pET-28a(+)/lacZ was transformed into *E. coli* DH5α using heat shock at 42 °C for 60 s to confirm the recombinant plasmid. Next, the stepwise deleted *vafklB* promoter regions were amplified using NB10 chromosomal DNA as the template and complementary primer sets containing the BglII and BamHI restriction sites (PCR condition: 25 amplification cycles of 97 °C for 30 s, 58 °C for 30 s, and 72 °C for 30 s). The PCR products and the purified pET-28a(+)/lacZ were digested using BglII and BamHI restriction enzymes, treated with ligase, and transformed again into *E. coli* DH5α to complete the VApro/wild, VApro/del1, VApro/del2, and VApro/del3. The *vafklB* promoters, which underwent step-by-step deletion, were checked using PCR with the primers used for each cloning, and they were confirmed to have suitably sized PCR products: 235 bp, 142 bp, 120 bp and 75 bp (data not shown). Additionally, the insertion of each target was confirmed via DNA sequencing with the Applied Bio-systems 3730XL using the BigDye(R) Terminator v3.1 Cycle Sequencing Kit (Applied Biosystems, Foster City, CA, USA)^68^. The construction process overview of the pVApro recombinant plasmid is presented in Fig. S2, and all primer sets used in this study are listed in Table 4.

**Table 4:** Primers used in this work.

Target regions and genes	Primer name	Oligonucleotide sequence (5ʹ→3ʹ)	Primer size (bp)
253 bp of the 5ʹ untranslated region of the *vafklB* gene; for molecular cloning	VApro/wild FP	GGCC **AGATCT** CTTAA CCCGT CTCCT TTTCT G	31
	VApro/wild RP	GGCC **GGATCC** AAACA GTCAC TCTTT TTA	28
142 bp of the 5ʹ untranslated region of the *vafklB* gene; for molecular cloning	VApro/del_1 FP	GGCC **AGATCT** ATCTT GAACA TTTTT CT	27
120 bp of the 5ʹ untranslated region of the *vafklB* gene; for molecular cloning	VApro/del_2 FP	GGCC **AGATCT** ATCTG CGATT TGTCA CAA	28
75 bp of the 5ʹ untranslated region of the *vafklB* gene; for molecular cloning	VApro/del_3 FP	GGCC **AGATCT** CATCC CATGC TTGAA	25
*vafklB* promoter region in recombinant plasmids; for verification of the cloning	VApro-seq FP	CTCAA GACCC GTTTA GAGGC	20
	VApro-seq RP	CCACG CCGAA ACAAG CGCTC	20
*lacZ* gene in pcDNA3.1/His/*lac*Z; for molecular cloning	lacZ FP	GGCC **GGATCC** ATGAT AGATC CCGTC GTT	28
	lacZ RP	GGCC **CTCGAG** TTATT TTTGA CACCA GACCA	30
*lacZ* gene in the recombinant plasmids; for qRT PCR	qRT-LazZ FP	GAGTG CGATC TTCCT GAGG	19
	qRT-LacZ RP	TCTGG CCTTC CTGTA GCCA	19
16 S rRNA of *E*. *coli* DH5α; for qRT PCR	qRT-16 S-EC FP	GACTC CTACG GGAGG CAGCA	20
	qRT-16 S-EC RP	GTATT ACCGC GGCTG CTGG	19
16 S rRNA of *V*. *anguillarum* NB10; for qRT PCR	qRT-16 S-VA FP	GTGCC AGCAG CCGCG GTAA	19
	qRT-16 S-VA RP	GACTA CCAGG GTATC TAATC	20

*FP* forward primer, *RP* reverse primer.

Bolds indicates restriction enzyme sites. AGATCT, BglII; GGATCC, BamHI; CTCGAG, XhoI.

### Measurements of promoter strength

#### β-Galactosidase assay

The β-galactosidase assay was performed according to the Miller method with minor modifications to quantify the activation level of the promoter in terms of number of Miller units of enzyme activity^69^. Briefly, 3 mL of overnight cultured recombinant *E. coli* DH5α harboring pVApro/wild (*E. coli* pVApro/wild) was inoculated into LB-Kanamycin (50 µg/mL) broth (300 mL). The cells were cultured for 6 h at 37 °C until the late log phase was 2 × 10^9^ CFU/mL. The cells were collected through centrifugation at 6,000 rpm for 10 min and immediately suspended in 50 mL of fresh LB medium, which was adjusted to pH 5–10 with 6 N HCl and NaOH. The samples for promoter strength measurement were collected after 1, 2, and 4 h of incubation at 25 °C. To measure promoter strength in response to temperature stress, the samples were incubated at 37 and 50 °C and pH 7, followed by centrifugation at 12,000 rpm for 10 min to remove the supernatant completely. The collected cells were resuspended in 50 mL of 50 mM Tris–HCl (pH 7.0 ± 0.2) buffer and disrupted using a sonicator (Sonics & Materials, Inc., Newtown, CT, USA) at 4 °C (3 s pulses at 150 W for 30 min with 2 s gap between pulses). After centrifugation, the supernatant was used as crude enzyme for determining β-galactosidase activity. Each sample was assayed for β-galactosidase assay with o-nitrophenyl-β-d-galactoside (ONPG) as the substrate as described with minor modifications^70^. The assay mixture (100 µL) containing 5 mM ONPG and crude enzyme solution were incubated for 10 min at 40 °C. The reaction was stopped by adding one volume of 1 M Na_2_CO_3_. The optical density of the reactants was read using a microplate reader (KLAB, Daejeon, Republic of Korea). The Miller formula was used to calculate the Miller units of enzyme per minute per milliliter of the sample. Each sample was processed in triplicate, and the average values were used to calculate the Miller units of the enzyme in each sample.

$$\:1\:\text{M}\text{i}\text{l}\text{l}\text{e}\text{r}\:\text{U}\text{n}\text{i}\text{t}\hspace{0.17em}=\hspace{0.17em}1000\:\text{*}\:\frac{({Abs}_{420}-\left(1.75\text{*}\:{Abs}_{550}\right))}{\left(t\text{*}v\text{*}\:{Abs}_{600}\right)}$$

where Abs_420_ indicates the absorbance of yellow o-nitrophenol; Abs_550_ indicates the scatter from cell debris; *t* indicates the reaction time in minutes; *v* indicates the volume of culture assayed in milliliters; and Abs_600_ indicates cell density.

#### Total RNA extraction and cDNA synthesis

Cultured bacteria were centrifuged at 12,000 rpm for 10 min, and the pellet was re-suspended in 1 ml TRIzol reagent (Invitrogen Life Technologies, Burlington, Canada) to isolate the total RNA. Subsequent preparation and washing were performed using the Hybrid-RTM RNA Isolation Kit (GeneAll Biotechnology, LTD, Seoul, Korea). DNA was hydrolyzed using RQ DNase I (Promega, Madison, WI, USA). Complementary DNA (cDNA) was synthesized using PrimeScript 1st Strand cDNA Synthesis Kit (Takara, Otsu, Japan) under the following conditions: initial reaction with random hexamer at 30 °C for 10 min, followed by extension at 42 °C for 60 min. The RNA and cDNA were quantified using NanoDrop ND-1000 spectrophotometer (Thermo Fisher Scientific, Waltham, MA, USA).

#### Gene expression analysis

To validate the in vivo differential expression of *vafklB* and *lacZ* in *V*. *anguillarum* NB10 and recombinant *E*. *coli* at the transcriptional level, quantitative real-time PCR (qRT-PCR) was performed using a Thermal Cycler Disc Real Time System Lite (model TP700/760, software version V5.0x) (Takara Bio Inc, Otsu, Japan) instrument and SYBR Premix Ex Taq (Tli RNaseH Plus, Takara, Kyoto, Japan)^71^. In brief, bacteria were collected under the appropriate conditions mentioned above, and total RNA was isolated from the samples following the manufacturer’s instructions using the GeneAll Hybrid-R RNA isolation kit (GeneAll Biotechnology Co. Ltd, Seoul, Korea). The Riboclear Plus kit (GeneAll Biotechnology Co. Ltd, Korea) was used to remove DNA from the isolated RNA. RNA purity and concentration were then assessed using a NanoDrop Lite spectrophotometer (Thermo Fisher Scientific, Wilmington, DE, USA). Subsequently, 1 µg of purified RNA was used to synthesize cDNA with the PrimeScript cDNA synthesis kit (Takara Bio, Inc., Kusatsu, Shiga, Japan). The two-step shuttle PCR protocol was optimized to include by 35 cycles of initial denaturation for 30 s at 95 °C, followed denaturation at 95 °C for 5 s and annealing and extension at 58 °C for 15 s. The PCR mixture (25 µL) contained 12.5 µL of 2x SYBR Premix Ex Taq, 0.5 µL of each primer (15 µM), 9.5 µl of sterile distilled water, and 2.0 µL of cDNA. The 16 S rRNA of both NB10 and *E*. *coli* was used as the housekeeping gene for internal control. The relative quantitative value was expressed in accordance with the 2^−∆∆Ct^ method^72^. All primers used are specified in Table 4, as previously mentioned.

### Statistical analysis

All data were subjected to one-way analysis of variance (ANOVA) using Statistical Package for the Social Sciences (SPSS), followed by Duncan’s multiple range test. Statistical significance was set at *p* < 0.05, unless otherwise noted.

## Electronic supplementary material

Below is the link to the electronic supplementary material.

Supplementary Material 1

## Data Availability

The whole genome sequence data supporting the findings of this study have already been deposited in the National Center for Biotechnology Information with the accession code PRJEB5701. The datasets used and/or analyzed during the current study are available from the corresponding author, Jong Min Lee, upon reasonable request.
