# Supplementary material for: Genetic characteristics of novel extreme alkaline-inducible promoter located in five prime upstream region of peptidyl-prolyl cis/trans isomerase from Vibrio anguillarum
Source: Sci Rep. 2025 Jul 1;15:21372. doi: 10.1038/s41598-025-98559-y (PMC12218036; doi:10.1038/s41598-025-98559-y)
Supplement: Supplementary file 1 — Supplementary Material 1 [file 41598_2025_98559_MOESM1_ESM.pdf]

Supplementary material

Genetic characteristics of novel extreme alkaline-inducible promoter located in five prime upstream region of peptidyl-prolyl cis/trans isomerase from *Vibrio anguillarum*

Dong-Gyun Kim[1,†], Gyu Min Kim[2,†], Dong Nyoung Oh[2], Young-Sam Kim[1], and Jong Min Lee[2,*]

[1] Biotechnology Research Division, National Institute of Fisheries Science, Busan, Republic of Korea; combikola@korea.kr

[2] Department of Biotechnology, Pukyong National University, Busan, 48513, Republic of Korea; jmlee84@pknu.ac.kr

[*]Corresponding author.

E-mail adress: jmlee84@pknu.ac.kr (J. M. Lee).

Tel: +82-51-629-5865

[†] These authors contributed equally to this work.

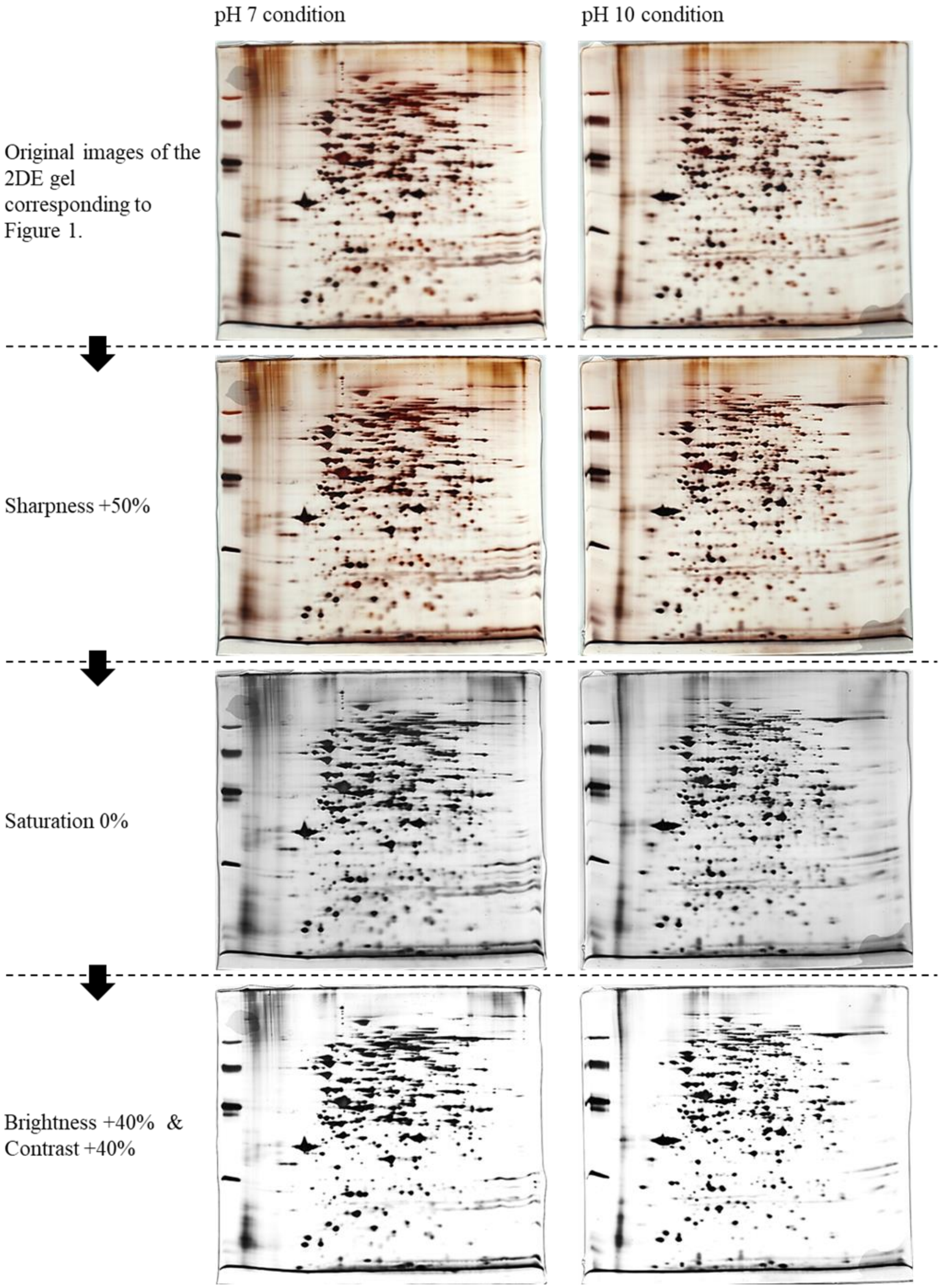

Figure S1. Original images of the 2DE gel corresponding to Figure 1, along with images from each adjustment stage.

23

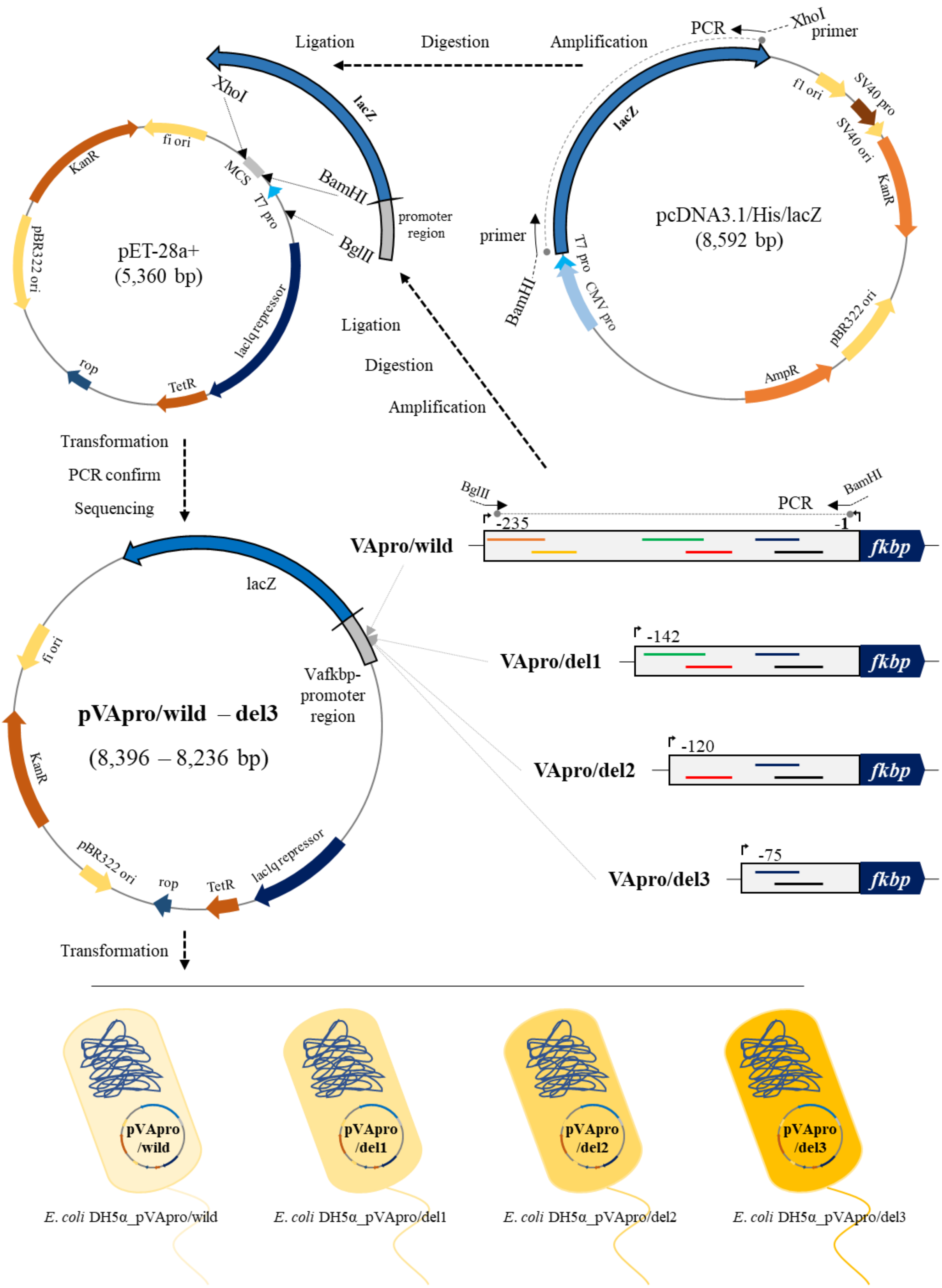

24 **Figure S2.** Schematic of the construction process for pVApro recombinant plasmids.
